# Tannin Fingerprinting in Vegetable Tanned Leather by Solid State NMR Spectroscopy and Comparison with Leathers Tanned by Other Processes

**DOI:** 10.3390/molecules16021240

**Published:** 2011-01-28

**Authors:** Frederik H. Romer, Andrew P. Underwood, Nadine D. Senekal, Susan L. Bonnet, Melinda J. Duer, David G. Reid, Jan H. van der Westhuizen

**Affiliations:** 1 Department of Chemistry, University of Cambridge, Lensfield Road, Cambridge CB2 1EW, UK; 2 Department of Chemistry, University of the Free State, 205 Nelson Mandela Drive, Park West, Bloemfontein 9301, South Africa

**Keywords:** vegetable tannins, polyphenols, chromium, aluminium, glutaraldehyde

## Abstract

Solid state ^13^C-NMR spectra of pure tannin powders from four different sources – mimosa, quebracho, chestnut and tara – are readily distinguishable from each other, both in pure commercial powder form, and in leather which they have been used to tan. Groups of signals indicative of the source, and type (condensed *vs*. hydrolyzable) of tannin used in the manufacture are well resolved in the spectra of the finished leathers. These fingerprints are compared with those arising from leathers tanned with other common tanning agents. Paramagnetic chromium (III) tanning causes widespread but selective disappearance of signals from the spectrum of leather collagen, including resonances from acidic aspartyl and glutamyl residues, likely bound to Cr (III) structures. Aluminium (III) and glutaraldehyde tanning both cause considerable leather collagen signal sharpening suggesting some increase in molecular structural ordering. The ^27^Al-NMR signal from the former material is consistent with an octahedral coordination by oxygen ligands. Solid state NMR thus provides easily recognisable reagent specific spectral fingerprints of the products of vegetable and some other common tanning processes. Because spectra are related to molecular properties, NMR is potentially a powerful tool in leather process enhancement and quality or provenance assurance.

## 1. Introduction

Tanning is an essential phase in one of civilization’s oldest processes, the transformation of hide and skins into leather [1], and vegetable tannins were probably the earliest used reagents [2]. In spite of the development of numerous synthetic fabrics, leather remains indispensible in many applications because of its distinctive properties: toughness, non-flammability, resistance to heat, impermeability to water, and permeability to air and water vapour. However, partly perhaps because the know-how built up over time is still able to produce satisfactory finished material, the fundamental molecular events underlying leather production are still not completely understood. Apart from the vegetable tannins, other reagents used in tanning include salts of chromium (III) and aluminium (III), and the bifunctional organic reagent glutaraldehyde [2]. Characterization of the nature of intermediates and the final product in any tanning process would clearly be of importance in mechanism-based attempts to enhance tanning efficiency or finished leather quality. Currently the industry uses certain standards based on the physical characteristics of intermediate and final product such as shrinkage and shrinkage temperature, and thermal properties, which give no direct clue as to any underlying chemical and physicochemical transformations. Detailed understanding of these could improve tanning methodology, finished product quality, and process control. It is also conceivable that chemical “signatures” could prove valuable in assessing and demonstrating the authenticity of high value leather products.

Nuclear magnetic resonance spectroscopy (NMR) is increasingly used in analysis and quality assurance of industrial samples of biological origin, such as fats, oils, and juices [3], material of wood origin [4], and biopharmaceuticals [5]. A major factor underlying this is that NMR seldom requires significant sample manipulation. In industry this is attractive because information-rich analyses can be carried out on raw materials, process intermediates or final products without possible confounding effects arising from sample manipulation, extraction, derivatization, or contamination. This can be a considerable advantage in internal process and quality control, and in presentation of data to external regulators. NMR has another significant strength: data is easily interpreted in chemical terms because each atom in a distinct environment gives rise to only a single signal, the frequency and characteristics of which are predictable consequences of molecular structure and environment. Thus many pure industrial substances, mixtures and composites produce unique and readily recognised spectroscopic “signatures”. Moreover with certain simple precautions signal intensity can be related to molecular abundance in a way not possible with more sensitive vibrational spectroscopy, mass spectrometry, and diffraction techniques. Finally, limited access to expensive NMR equipment is no longer the constraint to industrial applications that it once was, because high performance spectrometers are proliferating through academia and within certain industries themselves. 

NMR is useful in *in situ* characterization of tannic substances in wood derived materials [6], and changes in tannin content of woody materials under various transformation processes [7,8,9]. NMR can distinguish the solid tannins extracted from *Acacia mangium* [10] and maritime pine [11], and has been proposed as a general method of quantifying polyphenols, including tannins, in plant material [12,13]. Tannins leave a clear spectroscopic fingerprint in the ^13^C-NMR spectra of industrial materials in which they are used in adhesives [14,15], which moreover is sensitive to chemical changes brought about by processing [16]. NMR can also quantify the degree of extraction of tannins from bark [17], with the NMR results validating against conventional extractive gravimetric techniques.

Solid state NMR has also been used to study leather [18,19], and in a single case to infer tannin content [20]. The latter paper anticipates the general usefulness of NMR in characterization of leather tanned with vegetable tannins by emphasizing the convenient ^13^C-NMR spectral “window” between resonance frequencies of ca. 71 ppm and ca. 165 ppm which contains few signals from leather collagen protein but numerous signals from tannins. Tanning with other reagents has also been studied by NMR. Spectroscopic changes induced in model collagen peptides by Cr (III) [21] and Al (III) [22] are interpreted in terms of metal ion recognition by acidic protein groups. 

Here we extend this work to a systematic study of the effects exerted by vegetable tanning and other common tanning procedures on the NMR characteristics of whole leather, leading to the identification of spectroscopic changes characteristic of some of these. The study also identifies effects that certain tanning methods exert on the protein structures in leather, which may hold clues to the molecular events underlying different processes. 

## 2. Results and Discussion

### 2.1. Tanning with condensed tannins

**Figure 1:**
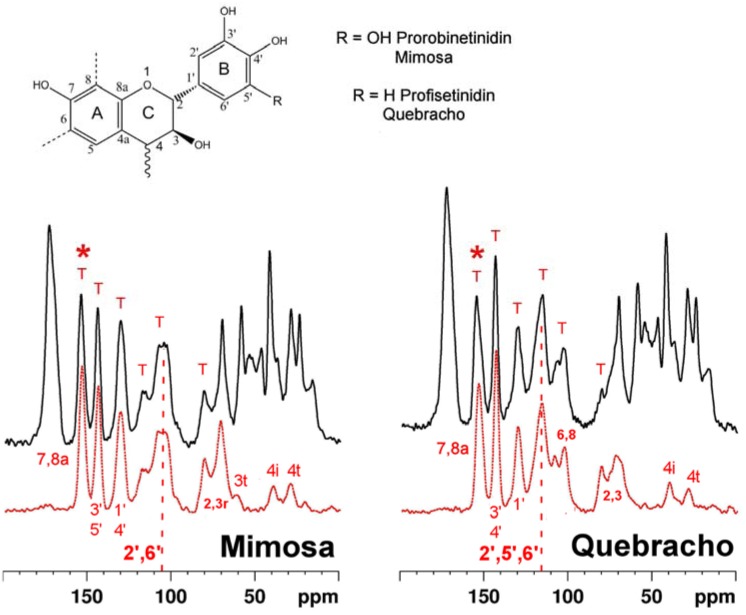
Solid state ^13^C CP-MAS spectra of leather (the black solid traces) tanned with tannins from mimosa **(left)** and quebracho **(right)**. These are plotted above identically acquired spectra from the respective pure tannins **(the red dotted traces)**. Also shown is a generic structure of the repeating flavonoid units predominating in each tannin; usually it is the A-ring 8, and 6, carbons (dashed bonds) which attach to the C-ring 4-carbon atom of a neighbour flavonol. In the spectra of the leather, tannin signals which are well resolved from collagen signals, and therefore reliable “fingerprints” of the tannin type used in the tanning process, are marked “T”. The vertical dashed lines highlight the envelope of signals the intensity of which is diagnostic of mimosa tanning (B-ring 2’ and 6’ carbon atoms at 105 ppm) and quebracho tanning (B-ring 2’, 5’ and 6’ carbon atoms at 115 ppm). Asterisks highlight signals from the A-ring 7 and 8a carbons indicative of condensed polyflavonoid tannins and their presence in leather. Some other assignments are shown; the suffixes “t” and “i” respectively indicate signals from carbon atoms at the terminus of, or internal to, the polyflavonol structure.

Spectra of leathers tanned with two types of condensed tannin, from mimosa and quebracho species, are shown in Figure 1. Each is accompanied by a spectrum of the respective pure tannin. Condensed tannins are complex oligomers of flavan-3-ol monomers of general structures as shown in the figure. The spectra of both pure condensed tannins are consistent with structure and with literature assignments of whole tannins and constituent monomers [23,24,25,26]. Moreover the condensed tannins from the two species are clearly distinguishable by their ^13^C-NMR spectral fingerprints. Most importantly each tannin spectrum is faithfully recapitulated in the spectra of the leathers tanned by them. Mimosa tannin is predominantly a prorobinetinidin polymer (resorcinol type A-ring and pyrogallol type B-ring) [27,28] while quebracho tannin is predominantly a profisetinidin (resorcinol type A- and B-ring). The B-ring C-H carbons (2', 5', 6') of quebracho tannin resonate conspicuously at ca. 115 and those of mimosa (2', 6' carbons) at 105 ppm, and are effective diagnostics of condensed tannin type in whole leather. The envelope of signals in which these markers are dominant are highlighted by vertical dashed lines in Figure 1.

### 2.2. Tanning with hydrolyzable tannins

**Figure 2:**
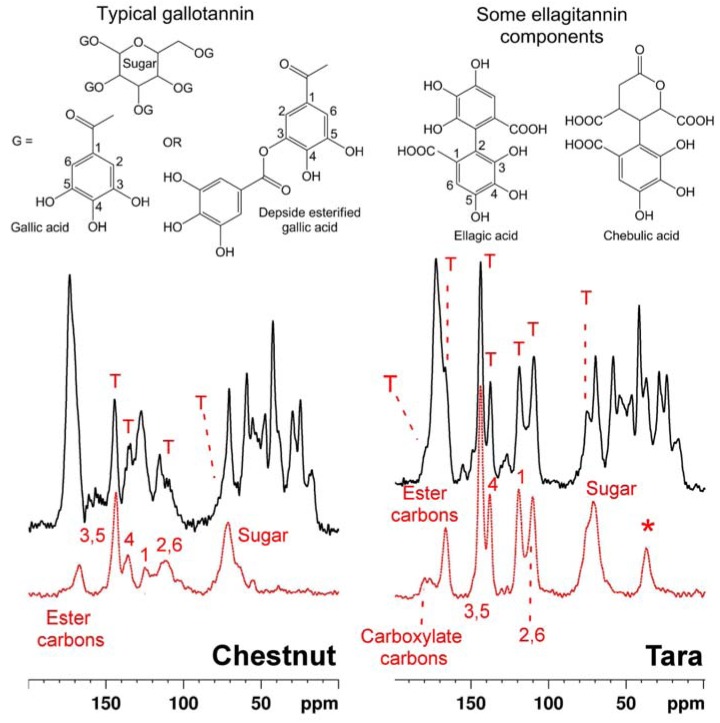
Solid state ^13^C CP-MAS spectra of leather tanned with tannins from chestnut **(left)** and tara **(right) (black traces)** plotted above spectra of the pure tannin corresponding to the respective leather sample. Also shown are some chemical structural formulae of typical constituents of hydrolysable tannin, and spectral assignments. The asterisk (*) indicates a signal due to non-sugar sp^3^ carbons in compounds such as the chebulic acid depicted here.

Similarly, spectra of leathers tanned with two hydrolyzable tannins, from chestnut and tara, are shown in Figure 2. Hydrolyzable tannins are complex mixtures of sugar monomers or polymers esterified with polyphenols such as gallic acid and its derivatives and closely related compounds [29,30,31]; typical constituent structures are exemplified in the figure. 

As in the condensed tannin cases, pure hydrolyzable tannin spectra reflect the chemistry of each [32,33,34,35]. Again the fingerprint of each tannin manifests itself clearly in the spectrum of each respective tanned leather. Moreover comparison with the leather spectra in Figure 1 shows that NMR convincingly differentiates between leathers tanned with condensed and leathers tanned with hydrolyzable tannins. The well resolved strong signal from the 7 and 8a carbons of the flavonoid A-ring at ca. 160 ppm is particularly diagnostic of the condensed tannin structure. This reporter signal is asterisked in Figure 1.

In principle it is possible to measure leather:tannin ratios from solid state NMR spectra and thereby infer the tannin load in the product. When cross polarization [36] from ^1^H to ^13^C (the standard ^13^C solid state observation technique) is used to enhance the inherently weak ^13^C signal, relating signal intensity to absolute molecular abundance is complicated by the fact that cross polarization efficiency is a function of molecular structure and must be established for different signals by examining their intensity dependence on cross polarization times. Although we have not carried out the necessary detailed studies it is nevertheless possible on the basis of a few simple assumptions to infer approximate leather collagen:tannin ratios from the data as presented here. If one assumes that cross polarization dynamics of non-protonated carbons are likely to be approximately similar to eachother, the well resolved signal from collagen amide carbons at ca. 175 p.p.m. is a measure of protein content, and the signal at ca. 160 p.p.m. (quaternary 7 and 8a carbons) is a measure of condensed tannin content. The ratio of the integrals of these two signals for both the mimosa and quebracho tanned materials is ca. 0.4 implying a molar ratio of collagen amino acid residues:tannin repeat units of ca. 0.2. Assuming an average molecular mass of 100 and 200 for collagen amino acid residues and tannin monomers, respectively, this molar ratio corresponds to a mass ratio of collagen:tannin of ca. 0.4. Quantification of hydrolysable tannins in leather is complicated by overlap between tannin carboxylate and ester carbons and collagen amide carbons, but tannin loadings seem to be quite similar to those resulting from the condensed tannin processes. 

Once the details of cross polarization dynamics have been established for a given material, however, more accurate quantification would be straightforward. Quantification by NMR will probably prove a more robust measure of tannin loading than other spectroscopic, or extractive, approaches, and likely informative in studies correlating leather properties with tannin loading and type. NMR should also yield useful information about the molecular events underlying processes using multiple reagents such as chrome, tannins, and other metal salts and organic tanning agents. 

### 2.3. Chromium (III) tanning

**Figure 3:**
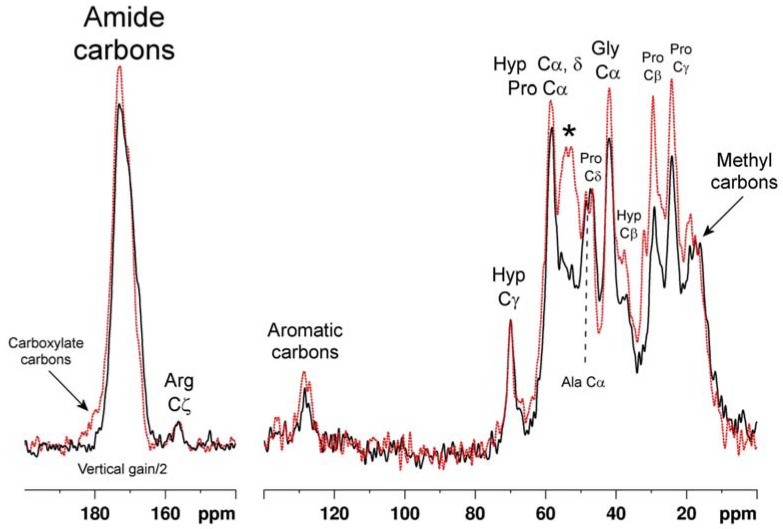
Solid state ^13^C CP-MAS spectrum of leather tanned with chromium (III) **(the black solid trace)** superimposed on a spectrum of untanned hide **(red dotted trace)**. Some assignments to carbon atoms in specific collagen amino acid residues or residue types are also indicated. The asterisk marks the envelope of overlapped signals from *inter alia* the α-carbons of several types of residues, including aspartate and glutamate. Ala – alanine, Gly – glycine, Hyp – hydroxyproline, Pro – proline.

Having shown that NMR was able to clearly recognize different vegetable tanning agents in final product materials, it was of interest to compare these leathers with leathers produced by processes which use other reagents. In Figure 3, a ^13^C-NMR spectrum of chromium (III) tanned leather is superimposed on that of untanned hide. The latter is very similar to that of pure collagen, and assignments [37] of some signals to specific amino acid residues or functional groups are shown. The ^13^C spectrum of the Cr (III) tanned leather is broadly similar, but it is notable that a number of signals in this spectrum are reduced in intensity relative to their counterparts in the native material. Cr (III), with three unpaired 3d electrons in its outer valence shell, is strongly paramagnetic. The large electron magnetic moment associated with the paramagnetic state can increase NMR relaxation rates, and/or shift the NMR frequencies, of nuclei which are close in space to the paramagnetic centre [38]. In the former case (relaxation enhancement) signals from atoms close to the paramagnetic centre broaden, and can become so broad that they actually become unobservable. It is very likely that the loss of signal intensity in the Cr (III) tanned leather is due to signals from atoms close to hydrated chromium ions bound into the collagen matrix thus becoming unobservably broad. Each signal in the collagen spectrum is due to overlap of a few, or numerous, signals from chemically equivalent, or similar, atoms in the large collagen molecule of ca. 1,000 amino acid residues, so it is impossible to ascribe chromium induced signal broadening to binding to any particular portion of the collagen structure. Moreover the precise magnitude of chromium induced signal broadening (or shifting) is a complex function of the geometric relationship of chromium ions and affected atoms, and of their sharing of unpaired paramagnetic electron density through direct bonding. For this reason it is impossible to pinpoint interactions at specific amino acid residues or locations on the collagen triple helix.

Nevertheless it is significant that there is marked broadening of the envelope of signals centred at ca. 53 p.p.m.; this region contains signals from the α-carbons of acidic aspartate and glutamate residues (among others). Also strongly broadened is the shoulder centred at ca. 180 ppm to high frequency of the amide carbon signal, comprising signals from the carboxylate carbons of the same acidic residues [21]. It is these acidic residues which are likely to play a significant role in binding Cr (III) into the collagen network.

### 2.4. Tanning with aluminium (III) and glutaraldehyde

**Figure 4:**
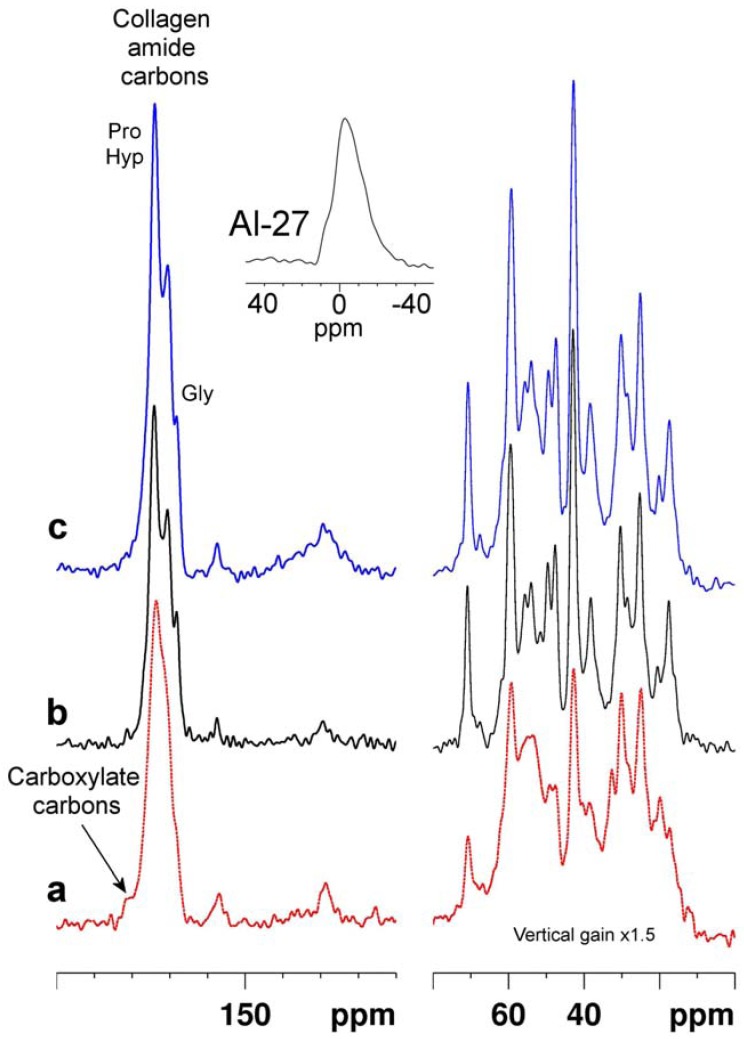
Solid state ^13^C CP-MAS spectra of untanned hide **(a, red dotted trace)**, and identically acquired spectra of aluminium sulphate tanned leather **(b, black trace), **and glutaraldehyde tanned leather in which the resolved backbone amide glycyl and prolyl/hydroxyprolyl signals are marked **(c, blue trace)**. The inset is a ^27^Al Hahn spin echo spectrum of the Al (III) tanned material.

Spectra of leathers tanned with aluminium sulphate, and with glutaraldehyde, are compared with a spectrum of an equivalent sample of untanned material in Figure 4. Also shown (inset) is an ^27^Al-NMR spectrum acquired from the Al (III) tanned material [39]. The chemical shift of the ^27^Al signal is close to that of aqueous hydrated Al^3+^ at 0 ppm, and the linewidth at half peak height is ca. 1.8 kHz. Neither the diamagnetic Al (III) ion nor the organic glutaraldehyde reagent produce the large changes in peak intensity caused by the paramagnetic Cr (III) ion. This is entirely to be expected because the ability of these diamagnetic species to exert long range effects on the NMR properties of the leather is much more limited than paramagnetic Cr (III). Nevertheless the response of the spectral linewidths of leather to tanning with either material is notable. Both tanned leathers exhibit signals which are generally much sharper than those of the parent untanned leather. In solid state NMR a significant cause of signal broadening is environmental heterogeneity resulting from partial or complete molecular disorder. Thus the signal sharpening seen with both processes is likely attributable to an increase in molecular order [40] brought about by whatever interactions, or reactions, occur between the tanning agents and the collagen matrix. These two chemically dissimilar reagents produce very similar changes in the collagen spectrum arguing for similar molecular ordering, although almost certainly *via* different underlying chemical processes. 

In the case of the Al (III) tanned leather the NMR properties of the ^27^Al isotope (100% natural abundance, gyromagnetic ratio, γ, about 1.04 times that of ^13^C) provide an extra NMR probe of the chemistry of the tanning process. The chemical shift of the ^27^Al signal argues for octahedral coordination by six oxygen ligands (as opposed to the other common tetrahedral geometry resulting from Al (III) coordination by four ligands) [41]. The quadrupolar nature of the ^27^Al nucleus with a nuclear spin I = 5/2 means that the ^27^Al resonance lineshape is responsive to electric field gradients at the nucleus, which are a function of its bonded and non-bonded environment, particularly the nature and symmetry of the inner and outer coordination spheres. The lineshape is potentially amenable to modelling in terms of Al (III) coordination environment and the distribution of these environments [42] but we have not attempted this. 

In the case of the glutaraldehyde tanned leather there are no clear signals attributable to the carbon atoms of the tanning reagent. There are several possible explanations; the incorporated glutaraldehyde molecules may be too mobile to cross polarize, their signals may be unobservably broad due to factors such as environmental heterogeneity, or the glutaraldehyde content required to produce a functional leather may simply be below the detection threshold of NMR. Much lower levels of reagent are used in the glutaraldehyde tanning process than in the vegetable tanning processes (see Section 3.1), probably reflecting the different molecular mechanisms of the covalently interacting aldehyde and the weaker non-covalently binding tannins. Additionally the signals of the covalently bound glutaraldehyde reaction products in the glutaraldehyde tanned leather would overlap with collagen signals further compromising the ability of NMR to detect and resolve them.

The effects of these two reagents, one a metal ion and the other a bifunctional reactive organic dialdehyde, on the spectrum of leather collagen are rather different from those of the vegetable tannins. While the sharpening of signals induced by Al (III) and glutaraldehyde is quite general throughout the leather collagen spectrum, it is best appreciated by considering the envelope of signals centred at ca. 175 p.p.m. due to the collagen amide carbons. In the Al (III) and glutaraldehyde tanned materials it is possible to resolve three distinct signals from amide carbons of (in order of increasing resonance frequency) glycyl, prolyl/hydroxyprolyl, and other, residues. This resolution is not observed in spectra of the untanned or vegetable tannin tanned materials, in which these three signals are broadened into a single envelope. 

## 3. Experimental Section

### 3.1. The tanning processes

UK bovine hides were prepared for tanning using standard industrial processes, as follows: hides were soaked, washed, limed to remove hair, fleshed to remove unwanted flesh, connective tissue and fat, split to a substance of 3.0 mm, and weighed. All further processing percentages were based on the limed weight. The hides were then washed, delimed with ammonium sulphate and formic acid to a pH of 8.3, bated to remove unwanted inter-fibrillary proteins, washed again and pickled using salt, and formic and sulphuric acids to a pH of ca. 2.8 (chrome), 3.0–3.3 (alum, glutaraldehyde) or ca. 4.5 (vegetable tannin extracts), and the penetration of pickle ensured to be complete. 

Hides were tanned with vegetable tannin extracts by adding 2% tannin and drumming for 30 minutes, followed by 10% tannin for 60 minutes, another 10% tannin (60 minutes) and a further 10% which was then drummed until penetrated. After tannage, a small percentage of EDTA (ethylenediamine tetraacetic acid) was added to sequester any iron present, followed by acidification with formic acid to a pH of 3.5. The leather was then given a light wash and dried (henceforth the leather is termed veg tanned). 

Chrome tanning was effected by adding 6–8% basic chromium sulfate (25% Cr_2_O_3,_ 33% basicity), running for 2–4 hours until penetrated and then basifying with magnesium oxide or soda ash or sodium bicarbonate to a pH of 3.8–4.2, followed by draining and washing (the resulting leather is now referred to as wet blue).

Hides were alum tanned by adding 4% salt and then 15% aluminium sulphate and running for ca. 5 hours, then adding 4% soda ash and running until penetrated. Glutaraldehyde tanning was carried out by adding 1.5–1.8% of masked glutaraldehyde and running for 5–8 hours until penetrated at pH 3.9–4.1. Hides were removed from the tannage bath without washing, piled and dried (the leather is now referred to as wet white). 

### 3.2. Solid state NMR

Dry leather samples were prepared for NMR by first cutting them into small pieces about 1 to 2 mm in size with a scalpel, and ball milling these for ca. 1 minute to a powder after freezing in liquid nitrogen, using a Sartorius Mikrodismembrator at 3,000 r.p.m.. Tannin powders were analyzed as received. All samples were packed into 4 mm outer diameter zirconia rotors (Bruker, Karlsruhe, Germany) and ^13^C-NMR spectra obtained using a Bruker AVANCE-400 9.4 Tesla wide bore spectrometer equipped with a standard dual channel broad band probe, at a magic angle spinning rate of 14 kHz and radio frequencies of 400.1 MHz (^1^H), 100.5 MHz (^13^C) and 104.2 MHz (^27^Al) respectively. ^13^C signal intensity was enhanced using standard cross polarization [36] (CP) MAS techniques (^1^H π/2 pulse length 2.5 μs, ^1^H cross polarization field 70 kHz, ^1^H-^13^C cross-polarization contact time 2.5 ms, broadband TPPM15 decoupling during signal acquisition at a ^1^H field strength of 100 kHz, recycle time 2 s, typical number of scans accumulated per spectrum ca. 3,000). Chemical shifts were referenced to the methylene signal from solid glycine at 43.1 p.p.m. relative to tetramethylsilane at 0 p.p.m.. ^27^Al spectra were acquired using a rotor synchronized Hahn spin echo (τ = 1 rotor period, 71 μs, π/2 pulse 3 μs, π pulse 6 μs, number of scans accumulated ca. 50,000) and 0.2 s recycle delay, and no broadband decoupling, which had been established to have no effect on the ^27^Al-NMR signal characteristics. Chemical shifts were referenced to the resonance of aqueous ca. 1 M AlCl_3_ in ca. 1 M HNO_3_ at 0 p. p. m.. 

## 4. Conclusions

Vegetable tanning agents all leave a distinctive spectroscopic signature in the tanned leather product by which the origin and type of the tannin used may be inferred. Several other commonly used tanning reagents also leave readily distinguishable spectroscopic fingerprints in leather products. Paramagnetic Cr (III) causes the loss of considerable signal intensity from the spectrum of the collagen proteins constituting leather without significantly changing the linewidths of remaining signals. The diamagnetic reagents Al (III) and glutaraldehyde cause considerable collagen signal sharpening indicative of an increase in collagen molecular order. It is to be expected that processes using mixtures of tanning reagents will leave equally distinctive fingerprints in the leather product. The fingerprint reflects not only the process chemistry but also underlying molecular mechanisms whereby tanning converts unprocessed leather into a commercial product. As such NMR potentially represents a powerful tool in understanding and improving tanning processes.
